# Exploration of the Synergy Between 2D Nanosheets and a Non-2D Filler in Mixed Matrix Membranes for Gas Separation

**DOI:** 10.3389/fchem.2020.00058

**Published:** 2020-02-05

**Authors:** Feng Shi, Junxia Sun, Jingtao Wang, Min Liu, Shaofei Wang, Xingzhong Cao, Zhikun Yan, Yifan Li, Suzana P. Nunes

**Affiliations:** ^1^Department of Chemical Engineering, Zhengzhou University, Zhengzhou, China; ^2^Biological and Environmental Science and Engineering Division, Advanced Membranes and Porous Materials Center, King Abdullah University of Science and Technology (KAUST), Thuwal, Saudi Arabia; ^3^Key Laboratory of Nuclear Radiation and Nuclear Energy Technology, Institute of High Energy Physics, Chinese Academy of Sciences, Beijing, China

**Keywords:** mixed matrix membrane, MXene, GO, HNTs, SiO_2_, Pebax

## Abstract

Dual-filler MMMs have attracted special interests in recent years because of the possibility of producing synergetic effect. This study is aimed at exploring the underlying synergy between two-dimensional (2D) nanosheets and a non-2D filler in mixed matrix membranes for gas separation. MXene or graphene oxide (GO) as typical nanosheet filler is selected to be in pair with a non-2D filler, SiO_2_ or halloysite nanotubes (HNTs), with Pebax as the polymer matrix. In this way, four pairs of binary fillers are designed and the corresponding four groups of MMMs are fabricated. By tuning the mass ratio of binary fillers, synergetic effect is found for each group of MMMs. However, the two 2D fillers found different preferential non-2D partners. GO works better with HNTs than SiO_2_, while MXene prefers SiO_2_ to HNTs. To be noted, GO/HNTs renders the membranes the maximum enhancement of CO_2_ permeability (153%) and CO_2_/N_2_ selectivity (72%) compared to Pebax control membrane, while each of them as single filler only brought about very limited enhancement of CO_2_ separation performance. The possible mechanisms are thoroughly discussed in terms of filler dispersion, nanosheet flexibility, and the tortuosity and connectivity of the surface diffusion pathways along nanosheets.

## Introduction

Mixed matrix membranes (MMMs), containing a continuous polymer phase and a dispersed inorganic filler phase was introduced by Kulprathipanja in 1980s (Kulprathipanja et al., 1988). The investigation of MMMs has been increasingly focused on solving the permeability-selectivity tradeoff of original polymer membranes since it aims to combine the advantages of inorganic materials with superior gas transport and good mechanical property, but also those of the polymer with the economic applicability and good machining performance (Li et al., 2013b; Rezakazemi et al., 2014; Vinoba et al., 2017; Wang et al., 2019b). Although MMMs have been introduced for many years, there is still plenty of room for development, because of its unique “4 M” characteristics including multiple interactions, multiscale structures, multiphase, and multiple functionalities (Li et al., 2013b), which have revealed infinite possibilities in designing and tuning the structure of membranes.

Currently, the research on MMMs mainly centers on the development and use of materials, the exploration of the membrane fabrication method and the study of the theoretical model for predicting the gas separation performance of MMMs (Vinh-Thang and Kaliaguine, 2013). Among these topics, the innovation of the materials and fabrication method mainly depends on the advent of new fillers with high selectivity, their distribution, and adhesion to the polymer matrix. The reported strategies in filler development can be categorized into the following four types: (i) *Exploration of new fillers*. During the past 20 years, various types of filler materials such as zeolites, metal organic frameworks (MOFs), covalent organic frameworks (COFs), SiO_2_, carbon nanotubes, graphene, etc., have been developed (Zornoza et al., 2011; Xin et al., 2015; Kim et al., 2016a; Vinoba et al., 2017; Idris et al., 2019). Besides the composition of the filler materials, their shape/morphology is important. In recent years, two-dimensional (2D) nanosheets such as graphene, graphene oxide (GO), MXene, molybdenum disulfide (MoS_2_), and graphitic-phase carbon nitride (g-C_3_N_4_) have been gaining increasing attentions due to the capacity of forming long-range tortuous channels in membrane, which hinders the diffusion of larger molecules but permits the transport of smaller ones (Smith and Freeman, 2014; Dong et al., 2016b; Zhang et al., 2019). (ii) *Chemical functionalization of existing fillers*. It is an extensively applicable strategy to overcome the poor interfacial compatibility between polymer and filler (Zhang et al., 2019), or to directly impart more efficient transport mechanisms—such as surface diffusion and facilitated transport—to membranes. (iii) *Creation of nanoscale morphologies on the filler surface*. Different from Chemical functionalization, this strategy was proposed to enhance the interfacial adhesion at the nanometer scale rather than molecular level, which is expected to reduce the possibility of interfacial rigidification. This strategy has proved valid for zeolites and other silicate fillers (Shu et al., 2007a,b; Bae et al., 2009), and it needs more attention when other molecular sieves are used as fillers. (iv) *Integration of dual fillers*. This strategy is usually easy to operate. The interaction between dual fillers and the matrix might improve their dispersion, providing different functional domains within a membrane. They might also provide a unique way to control the morphology of permeation channels (Wang et al., 2019a). As such, a synergy is likely to occur between dual fillers, and hence significantly improve the membrane performance.

Early studies involving dual fillers combined MOF (HKUST-1) and zeolite. The author demonstrated that the different natures of fillers could improve the dispersion and further increase the membrane performance due to a synergetic effect (Zornoza et al., 2011). In recent years, more and more dual-filler MMMs have emerged with fascinating phenomena (Tang et al., 2008; Zornoza et al., 2011; Hu et al., 2012; Galve et al., 2013; Valero et al., 2014; Li et al., 2015b, 2018; Ahmad et al., 2017; Jamil et al., 2019; Wong et al., 2019). Cornas' group has carried out a series of experiments to investigate the synergetic effect of two fillers with different natures (Galve et al., 2013; Valero et al., 2014). The silica-based MCM-41 as well as NH_2_-MIL-51 MOF was integrated into polysulfone or polyimide matrix. The resulting MMMs possessed enhanced permeability due to the mesoporosity of MCM-41, while the enhanced gas selectivity was originates from the microporosity and flexibility of MOF (Valero et al., 2014). Besides, the dispersion of MCM-41 was found significantly improved when MCM-41 was in combination with 2D JDF-L1 sheets, which was ascribed to the strong steric effect of JDF-L1 sheets. Meanwhile, the enhanced gas selectivity was interpreted by the preferential horizontal orientation of JDF-L1 sheets which hinder the gas transport of large gas molecules (Galve et al., 2013). Wu et al. incorporated carbon nanotubes (CNTs) and GO blesheets into Matrimid® matrix. The high aspect ratios and smooth walls of CNTs were thought to furnish fast gas permeation pathways, and the GO sheets were perceived as a selective barrier on account of the horizontal orientation and functional groups on the surface of GO. As a result, the MMMs exhibited super characteristics of both CNTs and GO in gas separation (Li et al., 2015b). As mentioned above, dual-filler MMMs can not only combine their advantages, but even lead to a synergy to acquire non-linear effects. Nevertheless, now only a handful of such studies can be found in the literature, demonstrating that dual-filler MMMs are still in the initial stage of development and the origin of synergetic effect needs further exploration.

**Figure 1:**
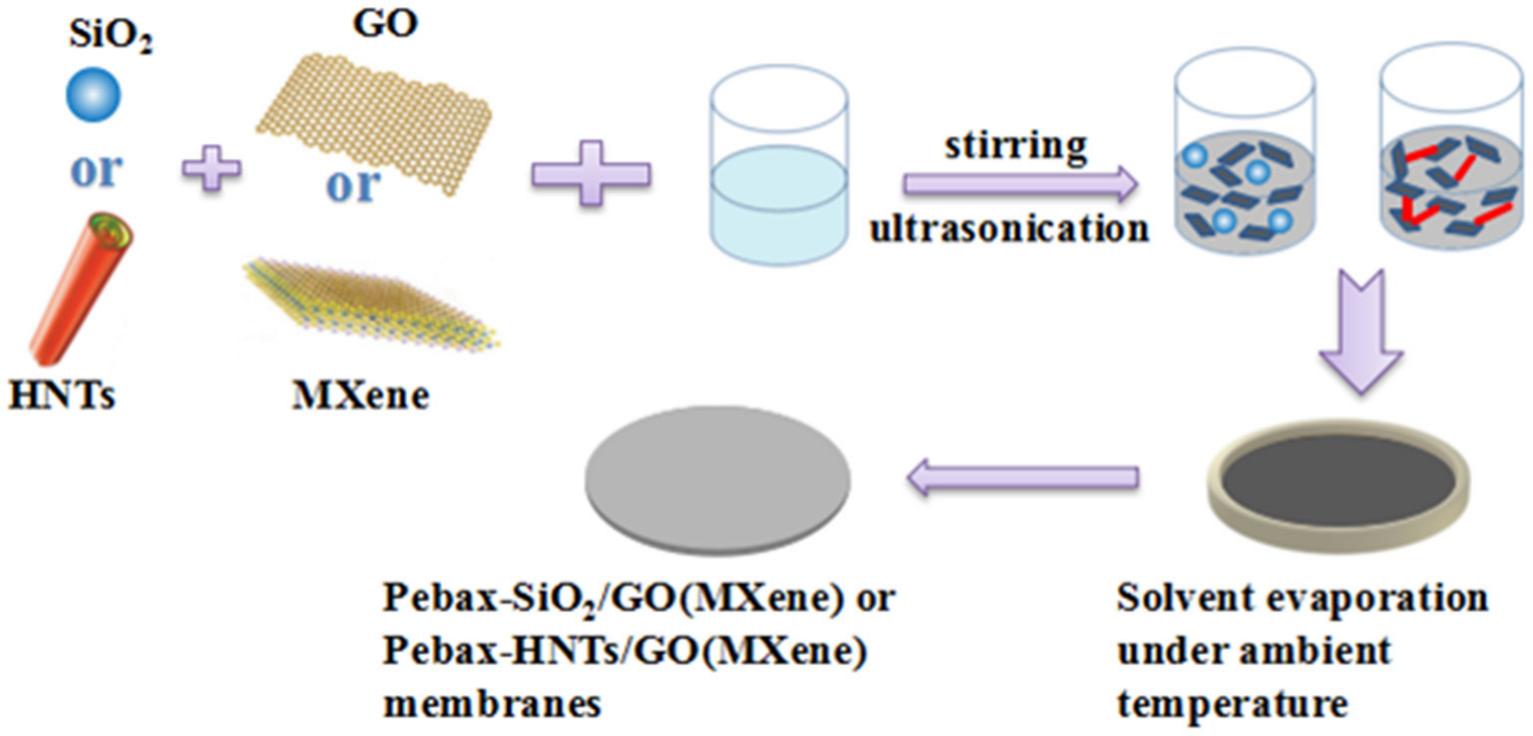
Scheme of the fabrication of dual-filler mixed matrix membranes.

In this study, we explored the synergy between 2D nanosheets and a second filler (1D or 3D) in MMMs. The 2D sheets were selected as the major filler, because they have distinct advantages in paving selective molecular pathways, but suffer from agglomeration and extra transport resistance (Zhang et al., 2019). GO and MXene were chosen as two representative 2D fillers for comparison with known differences of rigidity and surface functional groups (Jeon et al., 2016; Wang et al., 2018). SiO_2_ (3D) and HNTs (1D) were selected as the second fillers because of the highly controllable morphology and availability. GO/MXene was paired with SiO_2_/HNTs, resulting in four systems of dual fillers (Figure 1). The different matches were compared in terms of structures and gas transport properties, with the purpose of revealing part of the rules that could guide future work in this field.

## Experimental Section

### Materials

Commercial Pebax®1657 (consisting of 60 wt% PEO and 40 wt% PA_6_) was purchased from Arkema Inc. A mixture of ethanol and water (70/30 wt%) was used as a solvent for Pebax®1657. Ammonia water (NH_3_·H_2_O, 25%) and tetraethyl orthosilicate (Si(OEt)_4_, TEOS, 98%) were supplied by Feng chuan Chemistry Co., Ltd. (Tianjin, China) and Aldrich, respectively. Pristine HNTs were provided by Henan Xianghu Environmental Protection Technology Co., Ltd. (Henan province, China). Polystyrene sulfonate sodium salt (PSS, MW = 70,000) was provided by Sigma-Aldrich. Hydrofluoric acid (HF) and Ti_3_AlC_2_ powder were purchased from Sigma-Aldrich. Ethanol, hydrogen peroxide, hydrochloric acid, phosphoric acid, dimethyl sulfoxide (DMSO), sulfuric acid, and KMnO_4_ were provided by Kewei Chemistry Co., Ltd. (Tianjin, China). Deionized water was used throughout the experiment. The polymer and other chemicals were used as received without further treatment.

### Synthesis of Fillers

#### Synthesis of SiO_2_

Silica sub-microspheres were prepared according to the classical Stöber method (Chen et al., 2016): 2 mL of TEOS was added to the mixture of 200 mL ethanol, 20 mL deionized water and 15 mL aqueous solution of 25% ammonium with vigorous stirring at room temperature and the reaction was continued further for 24 h with stirring. The resultant silica particles were purified by three cycles of centrifugation, decantation, and resuspension in ethanol with ultrasonic-bathing. The silica particles were dried in a vacuum oven at 60°C till constant weight.

#### Modification of HNTs

Before modification, the pretreatment for pristine halloysite was required to obtain HNTs with uniform size. The pristine halloysite was mashed mechanically and soaked in deionized water for 2–3 days. Then the obtained slurry was filtered and dried in 50°C. Afterwards, the powder was grinded using mortar, and the HNTs were obtained after filtering through a 300-mesh sieve.

The HNTs was modified with PSS to improve their dispersion in Pebax®1657 (Qin et al., 2016; Zhang et al., 2018) 2 g PSS was dispersed in 100 mL deionized water in a flask, followed by 30 min agitation to form homogenous suspension. The resulting HNTs (2 g) were gradually added under continuous magnetic stirring for 48 h at ambient temperature and then left standing for 30 min to precipitate aggregates. The supernatant dispersion was collected and centrifuged at 5,000 rpm for 10 min and washed 3–4 times with deionized water until it became neutral. Finally, the obtained solid (PSS-HNTs) was dried in vacuum drier for 24 h and then ground into powder for use.

#### Synthesis of GO

The graphene oxide was synthesized through the improved Hummer's method as the literature reported (Zhang et al., 2019). Firstly, the suspended mixture solution of concentrated H_2_SO_4_/H_3_PO_4_ (540 mL/90 mL) was prepared in a 1,000 mL three-necked bottle, and then 4.5 g graphite powder and 27 g potassium permanganate were added into the mixture solution and stirred under 50°C for 24 h. The unreacted permanganate and manganese dioxide was transferred into soluble sulfates with 1,200 mL ice solution containing 10 mL H_2_O_2_ solution (30 wt%). The resulting suspension was re-dispersed by ultrasonic treatment and then centrifuged to separate the sediment, which was washed with the mixture solution of HCl/H_2_O (400 mL, 150 mL). The obtained suspension was stirred for 12 h washed with water until neutral, and was finally washed with ethanol followed by drying in the vacuum oven for 24 h.

#### Synthesis of MXene

MXene was synthesized following a previously reported method (Jeon et al., 2016). The Ti_3_AlC_2_ powder was etched with 49% HF aqueous solution under 60°C for 72 h to obtain the Ti_3_C_2_T_x_ sheets, which was added into DMSO solution for 48 h stirring to enable intercalation. With the purpose of exfoliation, plenty of water was added into the as-prepared solution and then centrifuged to separate the sediment. Finally, the obtained sediment was re-dispersed into water with a weight ratio of 1:500, followed by ultrasonic treatment and centrifugation to obtain the supernatant of MXene.

### Membrane Preparation

MMMs were prepared by a physical blending method. Firstly, a certain amount of Pebax®1657 was dissolved in ethanol/water mixture (70/30 wt%) with reflux under mild mechanical stirring at 80°C for 2 h to obtain 3 wt% homogeneous solution and cooled the solution to ambient temperature. Secondly, a certain amount of filler was fully dissolved into deionized water and added to the previously prepared polymer solution. After 30 min ultra-sonication treatment and 12 h stirring, the mixed homogeneous casting solution was poured onto Teflon Petri dishes. Eventually, the membranes were obtained after removing the residual solvent by drying at ambient temperature for 24 h. The thickness of membrane is in the range of 80–100 μm. The membranes were denoted as Pebax-A-X (A: GO or MXene) or Pebax-A/B-X/Y (B: SiO_2_ or HNTs), where X (0, 0.2, 0.5, 0.8, 1, 2.5, 5) denotes the wt% of filler A to matrix, and Y denotes the wt% of filler B to matrix.

### Characterization of Fillers and Membranes

The morphology was investigated by scanning electron microscopy (SEM) on Zeiss/Auriga FIB equipment, the membrane was broken in liquid nitrogen atmosphere, and all of the samples were cover with gold before observation. Besides, the transmission electron microscopy (TEM) was also used to investigate the morphology of membrane on a FEI Talos^TM^ F200S microscope. The chemical analysis was performed by Fourier Transform Infrared (FTIR) spectroscopy on a FTLA 2000 spectrometer in the 4,000–400 cm^−1^ scan range with resolution of 1.93 cm^−1^. The positron annihilation spectroscopy (PALS) analysis, which used 50 mCi of ^22^Na as the positron source, was used to measure the free volume of membranes. A GORTEC fast-fast coincidence system was used with the resolution of 201 ps.

### Gas Permeation Experiments

Membrane transport properties were measured by time-lag method. All the measurements were conducted under 2 bar and 30°C. The permeability coefficient *P* [Barrer, 1 Barrer = 10^−10^ cm^3^ (STP) cm cm^−2^ s^−1^ cmHg^−1^], diffusivity coefficient *D*, and solubility coefficient *S* of gas “i” were calculated by the following equations:

$$\begin{array}{l} P_{i}=\frac{V_{p}l\left(p_{p2}-p_{p1}\right)}{ART\Delta t\left(p_{f}-\left(p_{p1}+p_{p2}\right)/2\right)}\\ D_{i}=\frac{l^{2}}{6\theta }\\ S_{i}=\frac{P_{i}}{D_{i}} \end{array}$$

where *V*_*p*_ represents the constant permeate volume, *l* represents for the membrane thickness, Δ*t* is the time during which the permeate pressure increases from *p*_p1_ to *p*_p2_, *A* is the effective membrane area, and θ is the so-called time lag. The error of gas measurement is <10% for gas permeability and 20% for diffusivity coefficient, respectively.

## Results and Discussion

### Characterization of Fillers

#### The Physical Structure of SiO_2_, HNTs, GO, and MXene

**Figure 2:**
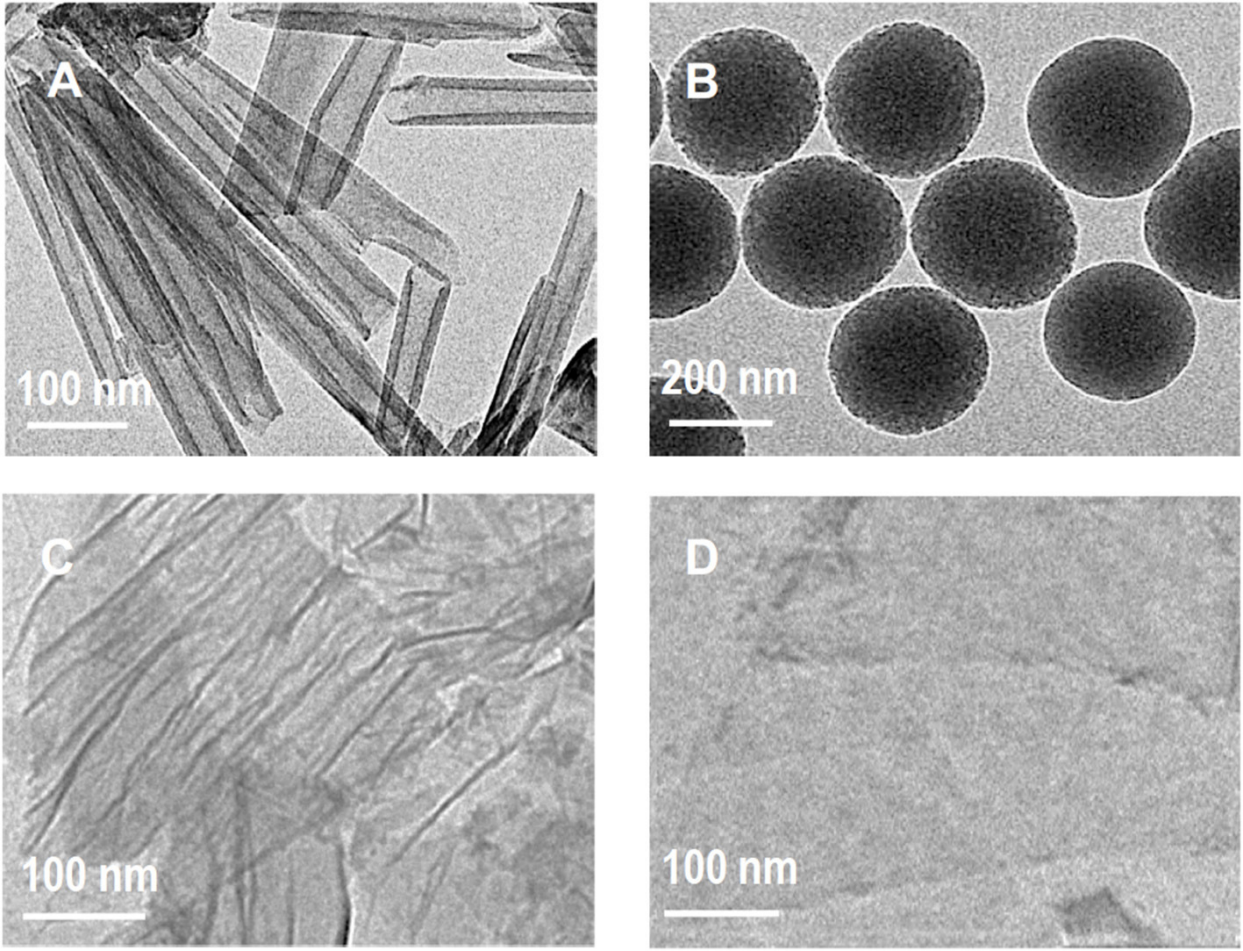
TEM images of **(A)** m-HNTs, **(B)** SiO_2_, **(C)** GO, and **(D)** MXene.

The physical morphology of the fillers was characterized by TEM, as shown in Figure 2. It's noticeable that HNTs has the inherent structure of hollow tubular and end-open structure. The tube length is 450–950 nm, the inner and external diameter is 20 and 44 nm, which is consistent with the literature (Qin et al., 2016; Liu et al., 2018). Figure 2B shows the relatively uniform and narrow particle size distribution with an average size of 200 nm for SiO_2_ microspheres. Both GO and MXene have the inherent sheet morphology of 2D-materials. Besides, it is obvious that GO and MXene are very thin. The lateral dimension and thickness of GO are 500–1,000 nm and 1.5–2.0 nm, respectively; for MXene, 1–2 μm and 1–2 nm. The TEM of GO sheets show wrinkles and folding edges, which are common in flexible GO sheets. On the contrary, the TEM of MXene sheets shows almost no wrinkles, which is expected in rigid MXene sheets (Shen et al., 2015).

#### The Chemical Structures of SiO_2_, HNTs, GO, and MXene

**Figure 3:**
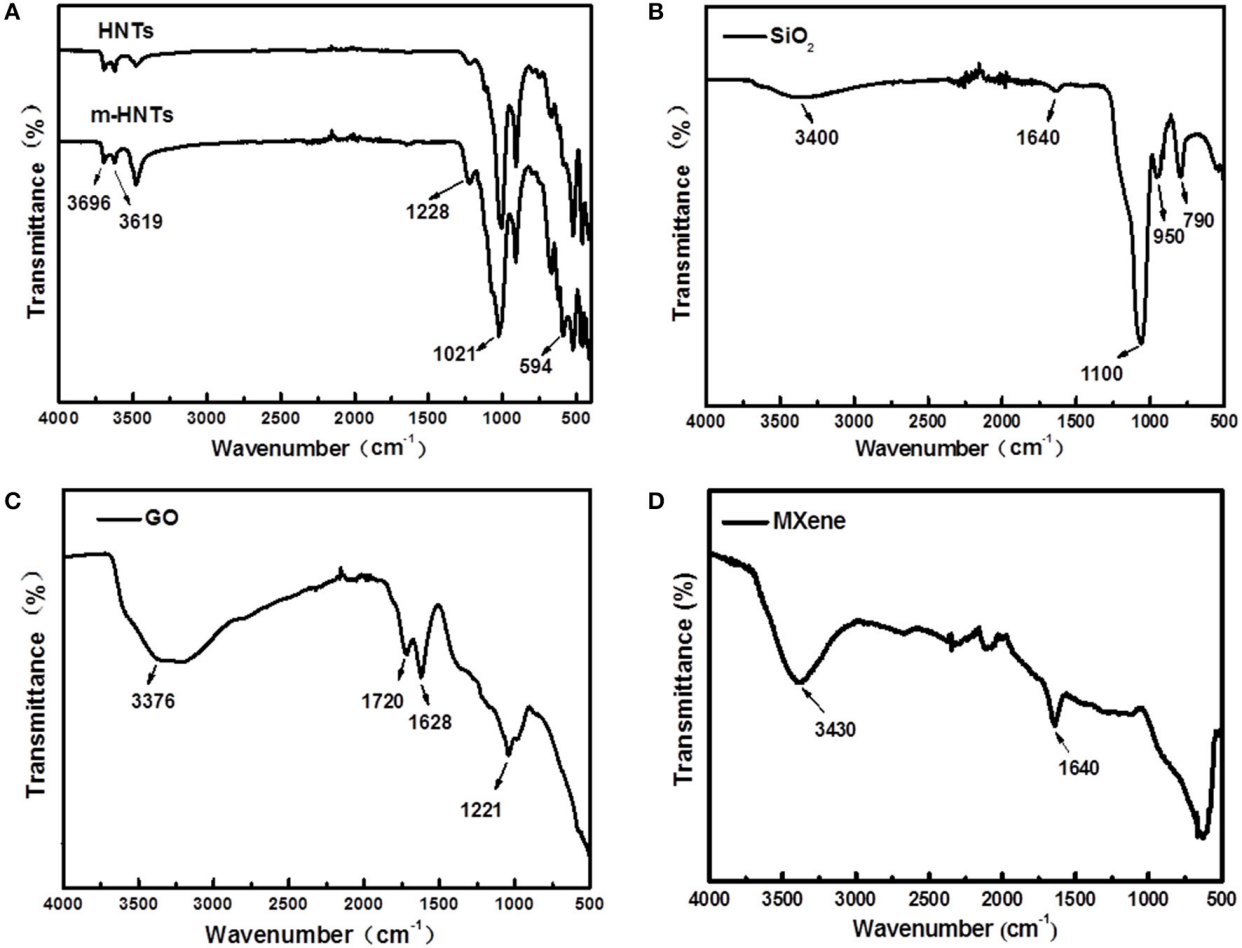
FTIR spectrum of **(A)** HNTs, **(B)** SiO_2_, **(C)** GO, and **(D)** MXene.

The chemical characteristics of the fillers were record by FTIR spectrum (Figure 3). In Figure 3A, the characteristic bands at 3,696 and 3,619 cm^−1^ correspond to the stretching vibration of hydroxyl groups of HNTs. The strong band at 1,021 cm^−1^ is assigned to the asymmetric flexible vibration of Si–O bond arising from the abundant O–Si–O groups in HNTs. Compared to the FTIR spectrum of pristine HNTs, the new and strengthened peaks at 1,228 and 594 cm^−1^ can be assigned to the asymmetric and symmetric vibration of S=O groups of –SO_3_Na (Qin et al., 2016). Figure 3B shows the FTIR spectrum of the SiO_2_ particles. The characteristic bands at 1,100 and 950 cm^−1^ are ascribed to the asymmetric stretching vibration of Si–O–Si and the stretching vibration of Si–OH, respectively (Li et al., 2014; Shi et al., 2019). Besides, the peaks at 1,640 and 3,400 cm^−1^ are the bending and stretching vibrations of water molecules bond to –OH groups of SiO_2_ (Kim et al., 2016b). In the spectrum of GO (Figure 3C), the band at 1,628 cm^−1^ corresponds to the sp^2^-hybrid carbon atoms. The bands at 1,221, 1,720, and 3,376 cm^−1^ are assigned to the C–O, C=O and C–OH, respectively, indicating that there are abundant oxygen-containing groups on the surface of GO (Quan et al., 2017). The MXene spectrum (Figure 3D) shows bands at 1,640 and 3,430 cm^−1^, which are assigned to the carbonyl group at the edge of MXene sheet and –OH stretching vibration, respectively (Gong et al., 2018).

### Filler Dispersion in Membranes

**Figure 4:**
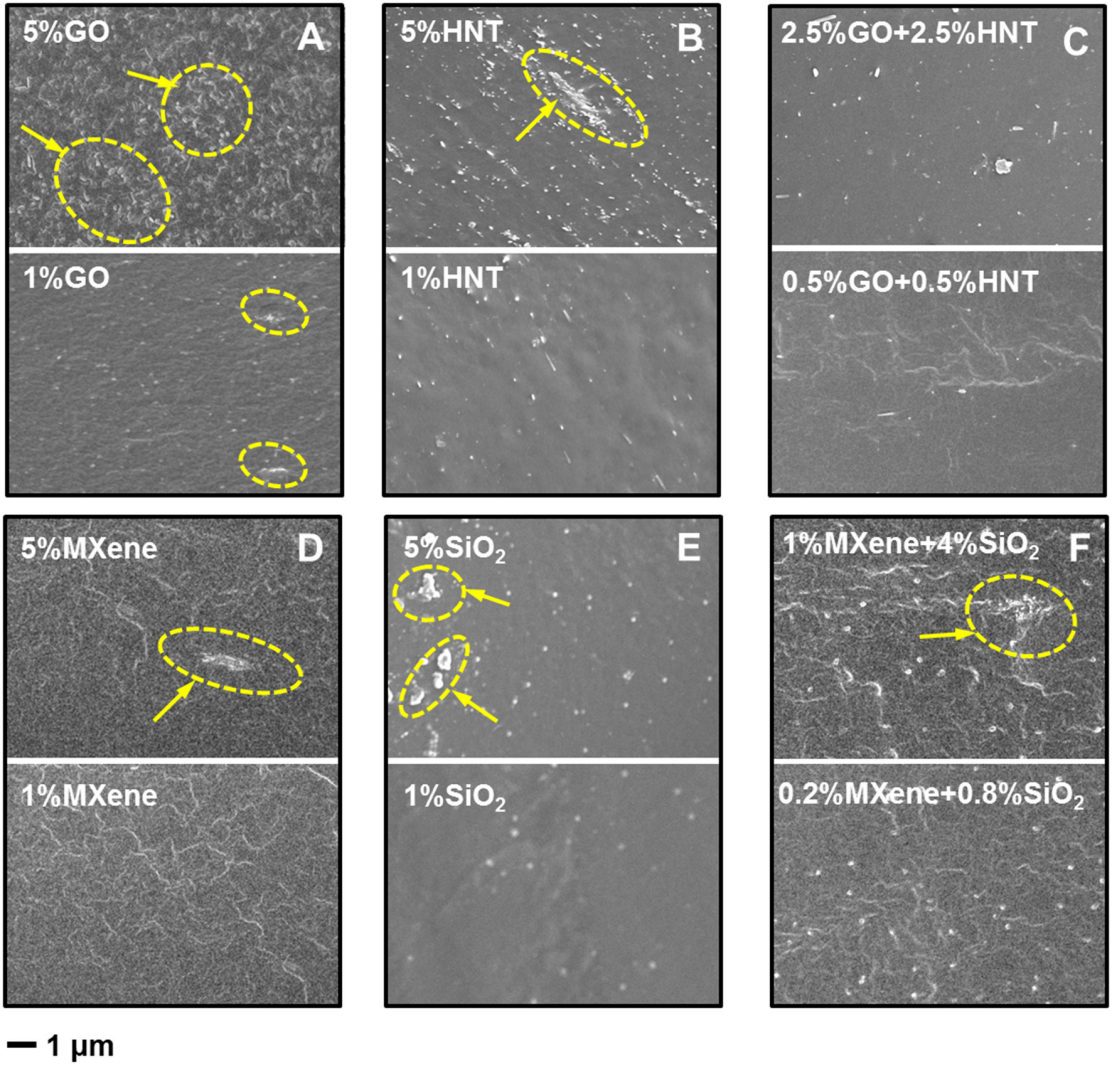
Cross-section SEM images of membranes filled with **(A)** GO, **(B)** HNTs, **(C)** GO+HNTs, **(D)** MXene, **(E)** SiO_2_, **(F)** MXene+SiO_2_. Circled area with arrows indicates aggregates.

The cross-section morphology of single filler MMMs, and dual-fillers Pebax-GO/HNTs and Pebax-MXene/SiO_2_ membranes, are shown in Figure 4 (with 1 and 5 wt% total filler content). It's noticeable that the dispersion of the high concentration (5 wt%) of 2D materials is not homogeneous (see aggregation in Figures 4A,D; Figure S1). Also, high concentration of HNT in Pebax suffers from severe agglomeration (Figure 4B) due to the high surface area. By comparison, the filler dispersion in dual-filler MMMs appears much better than each single-filler one. TEM image (Figure S2) confirms the good dispersion of both MXene and SiO_2_ in dual-filler membranes, especially at low total filler content (1 wt%). Meanwhile, compared to Pebax-GO membrane, the interfacial boundary between MXene and Pebax is more obscure, revealing that the dispersion of MXene might be more homogeneous than that of GO in membrane. This may results from the differences in functional groups of GO and MXene. MXene possesses higher density of functional groups (O, OH, and/or F) with more evenly distribution, which benefits an effective interaction between MXene and the Pebax matrix (Jeon et al., 2016). As for the cross-section image of Pebax-SiO_2_ membrane, the SiO_2_ achieves excellent dispersion in matrix at both 1 and 5 wt% content because of the small and uniform size, as illustrated in Figure 4E. However, when it comes to Pebax-HNTs membrane (Figure 4B), the HNTs exhibit inferior dispersion than SiO_2_, especially at 5% content, as a result of the extremely high aspect ratio and the strong van de Waals forces between HNTs (Wong et al., 2019). The dispersion is however better than for GO, probably due to some favorable interaction with the polyamide block of Pebax, as claimed before (Zhang et al., 2018).

Compared to Pebax-HNTs and Pebax-GO membrane, the Pebax-GO/HNTs membrane has an obscure HNT-polymer interface (Figure S3), indicating that there is synergetic effect between the mixed GO and HNTs that improve the dispersion of HNTs. Analogously to GO composites with carbon nanotubes, the flexible GO sheets could encase HNTs to facilitate the dispersion (Tian et al., 2010; Hu et al., 2012; Li et al., 2015b). On the other hand, the incorporation of HNTs would prevent the restack of GO sheet, which improves the dispersion of GO itself in matrix (Li et al., 2015b). Furthermore, there is a strong interaction between the surface functional group of GO and HNTs modified with sulfate. In addition to this, the membrane of Pebax-MXene/SiO_2_ also exhibits better interface morphology than Pebax-MXene membrane, revealing that the addition of SiO_2_ improves the dispersion of MXene sheets in the matrix. At the same time, the dispersion of SiO_2_ is not destroyed in dual-filler MMMs. Besides, there is also a strong hydrogen bond interaction between the hydroxyl groups of MXene and SiO_2_ and this further improves the filler dispersion (Hu et al., 2012).

### Gas Transport Properties of Membranes

**Figure 5:**
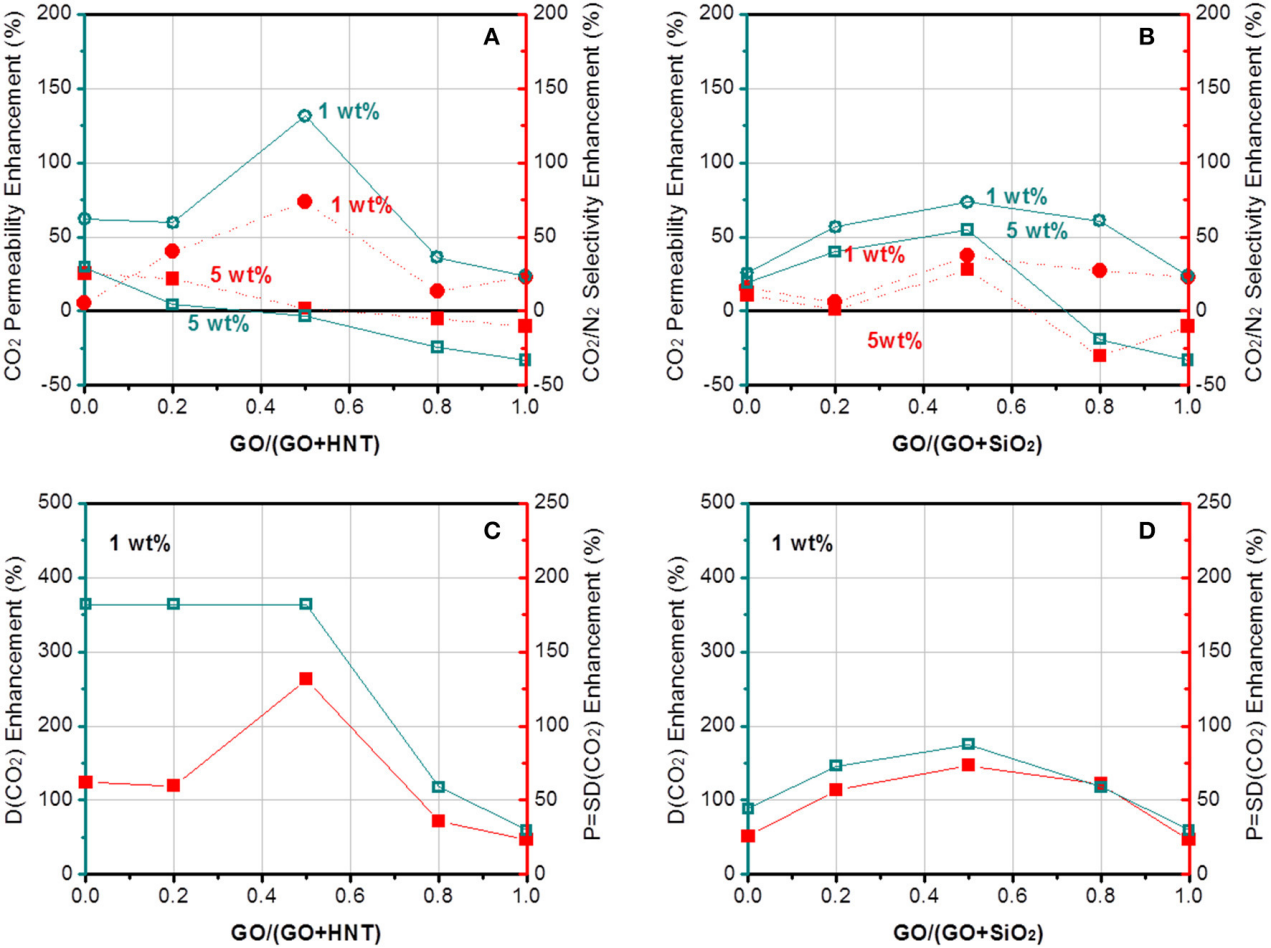
The enhancement of **(A,B)** CO_2_ permeability and CO_2_/N_2_ selectivity and **(C,D)** CO_2_ diffusivity of Pebax-GO/HNT and Pebax-GO/SiO_2_ membranes.

**Figure 6:**
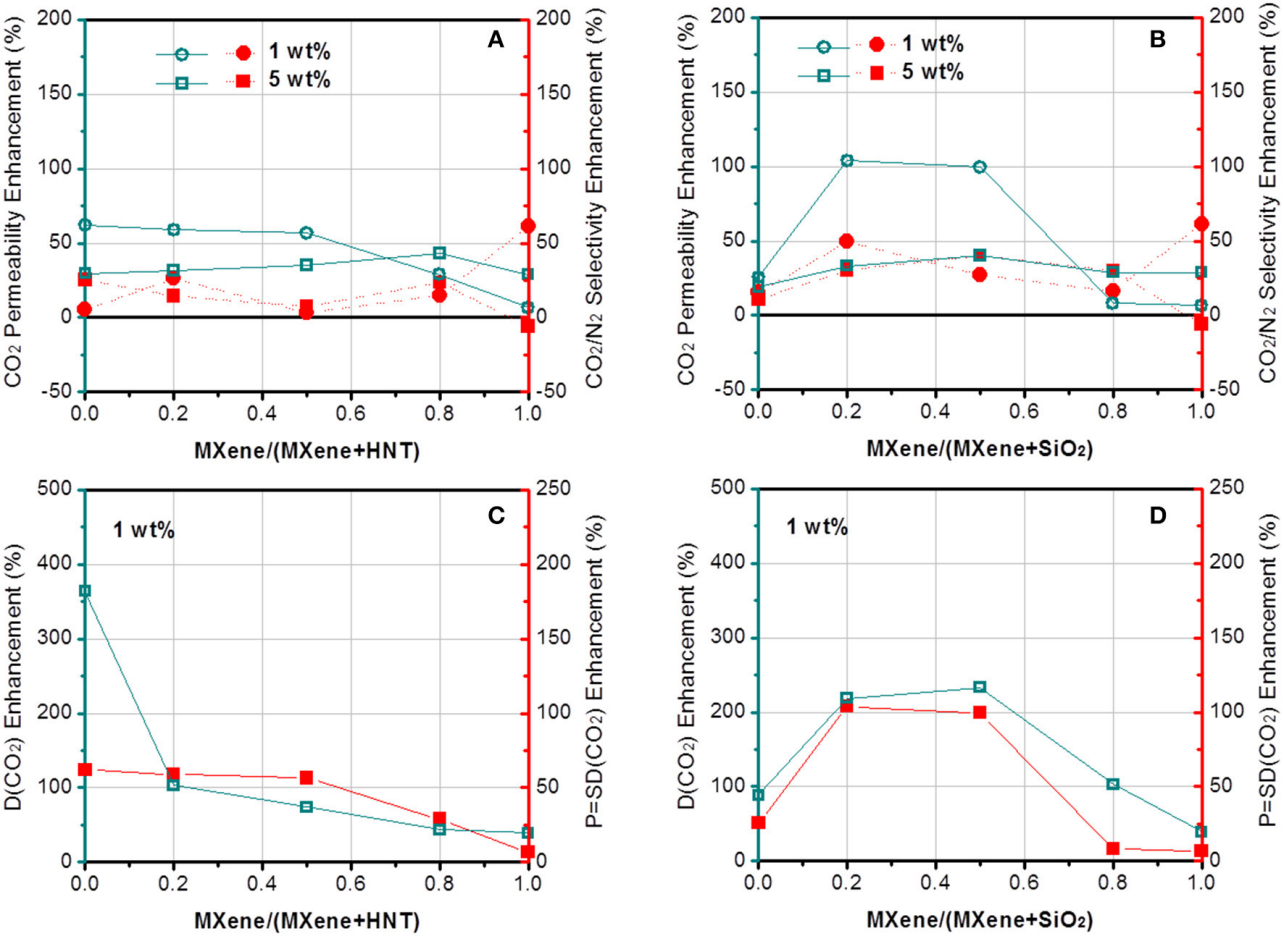
The enhancement of **(A,B)** CO_2_ permeability and CO_2_/N_2_ selectivity and **(C,D)** CO_2_ diffusivity of Pebax-MXene/HNT and Pebax-MXene/SiO_2_ membranes.

The gas separation performance of dual-filler MMMs based on GO and MXene was tested and shown in Figures 5, 6. CO_2_ permeability of 106 Barrer and CO_2_/N_2_ selectivity of 41 were measured for the pristine Pebax membrane. The effects of both overall loading (1 and 5 wt%) of fillers and the relative content of 2D fillers can be clearly seen (permeation data of membranes based on other overall loading can be found in Figure S4, which shows 1 wt% is the optimal overall loading). From a general view, we find a maximum in performance in different dual-filler MMMs, demonstrating the occurrence of synergetic effect. It is also notable that 1 wt% is usually the better overall filler loading than 5%, which accords well with the reported single-filler Pebax-based MMMs in the literature (Li et al., 2013a; Dong et al., 2016a). This also can be understood by considering the morphological observation in Figure 4. When a higher concentration of filler is added, aggregation occurs and the expected improvement is partially lost. Only a homogeneous distribution can lead to a significant performance enhancement. Since Pebax belongs to a solubility-controlled class of polymer, the major factor that determines the separation performance is the content and distribution of PEO domains, which preferentially interacts with CO_2_. Relatively small content of filler could disturb the PEO crystallization and increase the content of the amorphous PEO phase segments, making them more available to interact with CO_2_ (Yave et al., 2010), while excessively increasing the filler content would reduce the PEO content of the whole membrane with an undesirable aggregation, which does not effectively affects the crystallinity (Table S2). As a result, the series with overall loading of 1 wt% generally show more pronounced synergetic effect than those with 5% loading. This phenomenon is not as distinct for MXene-containing MMMs (Figure 6), which can be ascribed to a better dispersion of MXene than GO at high loading.

**Figure 7:**
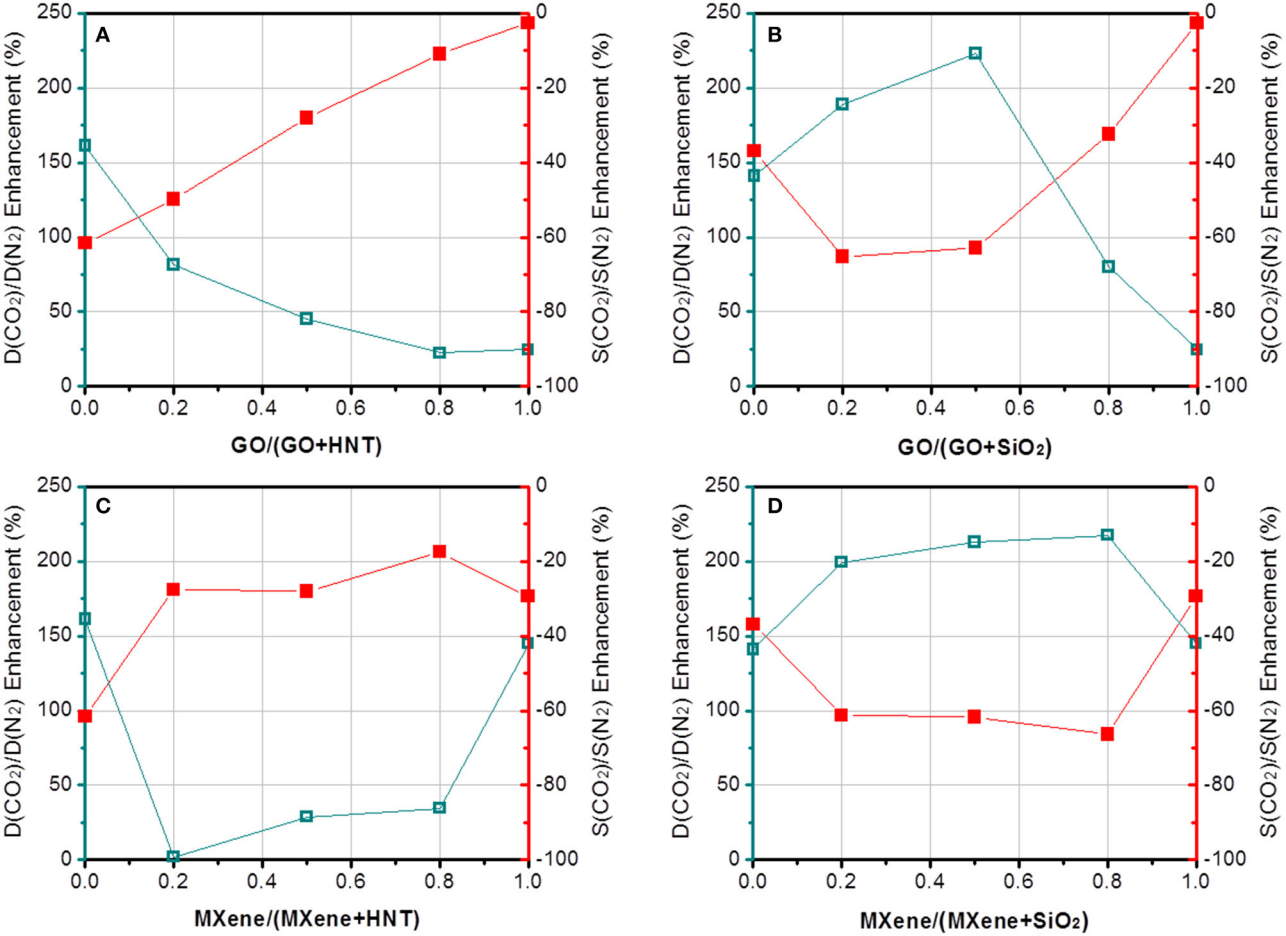
The enhancement of diffusivity selectivity and solubility selectivity of **(A)** Pebax-GO/HNT, **(B)** Pebax-GO/SiO_2_, **(C)** Pebax-MXene/HNT, and **(D)** Pebax-MXene/SiO_2_ membranes. The overall loading is fixed at 1 wt%.

More interesting results can be found by comparing the four pairs of dual fillers. First, GO is selected as the common filler and the effects of HNTs and SiO_2_ are compared. The results show that Pebax-GO/HNTs membranes has both higher CO_2_ permeability and CO_2_/N_2_ selectivity than Pebax-GO/SiO_2_ membranes, especially at 1 wt% overall filler loading. The Pebax-GO/HNTs-0.5/0.5 membrane exhibits optimal gas separation performance. The CO_2_ permeability and CO_2_/N_2_ selectivity achieve 131 and 74% enhancement, respectively, compared to the Pebax control membrane. On one hand, the enhanced CO_2_ transport originates in part from the ameliorative filler dispersion in the dual-filler MMMs, which increases the effective area of filler and thus leads to an enhanced CO_2_ transport of membrane. Besides, as shown in Table S1, Pebax-GO/HNTs exhibits higher gas diffusivity but lower gas solubility than Pebax-GO/SiO_2_ membrane, demonstrating that the SiO_2_ particle is much more CO_2_-philic than HNTs. Owing to the relatively lower diffusion resistance, Pebax-GO/HNTs (0.5/0.5) membranes exhibit 33% higher gas permeability than Pebax-GO/SiO_2_ (0.5/0.5) membranes. According to Figures 5C,D, the enhanced permeability compared to the pristine Pebax membrane is mainly based on the increase of diffusivity, particularly to CO_2_. The fillers are not enhancing the CO_2_ solubility, which in Pebax is already quite high and responsible for its excellent performance for CO_2_/N_2_ separation. The addition of fillers increased the CO_2_ diffusivity up to 364%, particularly in the case of the GO/HNTs dual filler system. Simultaneously, a maximum enhancement of CO_2_/N_2_ diffusivity selectivity considering diffusivity changes for both gases was higher than 130%. The enhancement of CO_2_/N_2_ selectivity can be understood from Figure 7, which clearly shows that the overall selectivity enhancement is determined by diffusivity selectivity, and the solubility selectivity of each MMM is lower than the pristine Pebax membrane. The increment of diffusivity selectivity is acquired at the cost of sacrificing solubility selectivity, and similar phenomena can be found in the literature, where GO was incorporated into Pebax membrane (Li et al., 2015a). It was reported that the complexation enthalpy of CO_2_-dimethyl ether complex is approximately 8 kJ mol^−1^ (Van Ginderen et al., 2003), reflecting the strength of typical dipole–quadrupole interactions. However, the hydrogen bonding energy for O–H…O was reported to be much higher (20~30 kJ mol^−1^) (Zhao et al., 2017). Therefore, it is reasonable to speculate that the hydrogen bonding between PEO and hydroxyl group-containing fillers will affect the formation of CO_2_-ether complex, which may decrease both the solubility coefficient and solubility selectivity. Another possible reason is that the presence of HNTs might induce the horizontal orientation for both GO sheets and HNTs, which is known to create a tortuous path to transport (Wong et al., 2019). In this way, GO/HNTs pair can effectively improve the tortuosity of gas transport channel, and hence the CO_2_/N_2_ selectivity (Li et al., 2015b). Decisive evidences can be found from the PALS data (Table S3). The notable decrease of *r*_3_ from 0.316 nm (Pebax-GO-1) or 0.317 nm (Pebax-HNTs-1) to 0.311 nm (Pebax-GO/HNTs-0.5/0.5) clearly reveals that the co-existence of GO and HNTs produces synergetic effect that increases the chain rigidity and diffusivity selectivity. This effect can be better understood though the interfacial morphology theory. Since HNT is mesoporous filler with lumen size up to 20 nm, there is no doubt that partial pore blockage by polymer chains will occur. Furthermore, in this study, the HNT was modified with PSS, which further enhanced the interactions between HNT and Pebax due to the favorable interactions between PSS and PEO chains (Wang et al., 2005; Mcdonald and Hammond, 2018). On the other hand, HNT can significantly enhance gas diffusivity due to the presence of broad internal channel, and Pebax-HNTs-1 membrane shows the highest CO_2_ diffusivity coefficient among all the membranes prepared in this study (Table S1). It is not surprising that the large inner diameter of HNT does not bring any decrease of permeability, considering the potential pore blockage interfacial morphology. According to the updated morphological diagram proposed by Ismail's group (Hashemifard et al., 2011), the best interfacial morphology for mesoporous filler-based MMMs is often “rigidification” or “pore blockage,” rather than the ideal case. The chain rigidification effect caused by fillers can be also seen from the transition temperature data shown in Figure S5. In this way, HNTs as fillers have the potential to simultaneously enhance permeability and selectivity. Although the concentration of HNT is rather low (1 wt%), the nanotubes are well-dispersed, especially at the presence of GO. The good dispersion of HNTs are beneficial to make full use of their benefits. When increasing the overall filler content up to 5%, the membranes exhibit decreased gas separation performance, which probably derived from the filler agglomeration that decreased the property of membrane.

In contrast to GO, MXene works better with SiO_2_ than with HNTs in matrix. The difference between Pebax-MXene/HNTs and Pebax-MXene/SiO_2_ is not as sharp as that between Pebax-GO/HNTs and Pebax-GO/SiO_2_, but the highest values of CO_2_ permeability and CO_2_/N_2_ selectivity of Pebax-MXene/SiO_2_ membrane are obviously higher than those of Pebax-MXene/HNTs membrane at 1 wt% overall loading. The Pebax-MXene/SiO_2_-0.2/0.8 membrane shows optimal gas separation performance with 104 and 49% enhancement of CO_2_ permeability and CO_2_/N_2_ selectivity based on pure Pebax membrane, or 129 and 119% as high as those of Pebax-MXene/HNTs-0.2/0.8 membrane, respectively. By comparing Figures 6C,D, there is continuous decline of CO_2_ diffusivity with the increase of relative content of MXene/HNT dual fillers, but the MXene/SiO_2_ dual fillers result in a maximum CO_2_ diffusivity higher than that of each of the corresponding single-filler MMM. That is, MXene/SiO_2_ dual fillers produce synergic effect while the MXene/HNT ones do not. Since SiO_2_ microspheres synthesized by Stöber method is known to achieve mono-dispersion, the dispersity of SiO_2_ is expected to much better than HNT, and the former is therefore envisaged to better interrupt the stacking of MXene nanosheets into thicker ones.

If we keep the HNTs concentration constant and compare the two 2D fillers, we can find that Pebax-GO/HNTs membrane shows much higher separation performance (especially CO_2_/N_2_ selectivity) than Pebax-MXene/HNTs membrane with the overall loading at both 1 and 5 wt%. Notably, the CO_2_ permeability and CO_2_/N_2_ selectivity of Pebax-GO/HNTs-0.5/0.5 membrane are 48 and 69% higher than those of Pebax-MXene/HNTs-0.5/0.5 membrane, respectively. Such results stem from the difference in rigidity between GO and MXene. In single-filler MMMs, HNTs tend to agglomerate in the matrix because of the high aspect ratio and strong inter-particle Van de Waals forces, thus cause sharp performance degradation (Wong et al., 2019), although after modification with PSS, a considerable improvement has been observed (Zhang et al., 2018). When it comes to dual-filler MMMs, the flexible GO sheets are known to be able to wrap the nanotubes and thus retard their agglomeration (Meng et al., 2012). Despite the lack of clear evidence of GO-wrapped nanotubes, the steric effect arising from GO sheets and the hydrogen interaction of surface functional groups between GO and HNTs can also promote the dispersion of HNTs, therefore improving the effective surface area of the fillers to furnish gas transport pathways. Furthermore, the preferential horizontal orientation GO and HNTs improve the tortuosity of gas transport, which increase the CO_2_/N_2_ selectivity. For Pebax-MXene/HNTs membrane, since MXene is more rigid than GO and more difficult to be dispersed as single sheets, the amelioration of HNTs dispersion is not pronounced.

From another perspective, MXene outperforms GO when SiO_2_ incorporation is fixed. Pebax-MXene/SiO_2_ membrane shows superior CO_2_ permeability and CO_2_/N_2_ selectivity compared to Pebax-GO/SiO_2_ membrane, which is distinct at 1 wt% filler content. Especially, the CO_2_ permeability and CO_2_/N_2_ selectivity of Pebax-MXene/SiO_2_-0.2/0.8 membrane is 104 and 49% enhanced compare to the pristine Pebax. Again, according to Figures 6D, 7 the enhancements are due to increase in diffusivity, since the CO_2_ solubility in the dual filler MMM is slightly smaller than in the pure polymer. Herein the *r*_3_ values from PALS data do not reveal the same chain rigidification effect as shown in Pebax-GO/HNT membrane. This phenomenon is reasonable because there is no pore blockage and PSS modification around SiO_2_ surface. The enhancement of diffusivity selectivity can be only interpreted by the tortuosity of gas transport channel. For the dual filler MXene/SiO_2_-0.2/0.8, there are two advantages for acquiring good MXene dispersion: the very low MXene concentration and the presence of highly disperse SiO_2_ microspheres. In this case the MXene nanosheets can create more diffusion obstacles and prolong the molecular diffusion routes, so as to effectively enhance diffusivity selectivity.

The effect of temperature and pressure on membrane performance was also investigated. As shown in Figure S6, each membrane displays a substantial increment when the operation temperature increases from 30 to 60°C, which typically represent the increase of gas diffusivity and polymer chain mobility. Interestingly, the decline of selectivity is not as distinct as the increase of permeability. Since solubility selectivity is very sensitive to temperature change, this fact further supports the diffusivity-dominated selectivity mechanism, and indicates the adequate polymer-filler interactions within the temperature range. In addition, the dependence CO_2_ permeability on temperature can be further correlated according to Arrenius relationship (Figure S7). The slope of each straight line is known to reflect the activation energy of CO_2_ permeation. As such, Pebax-GO/HNT-0.5/0.5 shows the lowest CO_2_ permeation activation energy, in good accordance with the high diffusivity coefficient at room temperature. Figure S8 shows that CO_2_ permeability as well as CO_2_/N_2_ selectivity only changes little in the pressure range from 1 to 5 bar, which accords well with the Pebax-based MMMs reported in the literature (Duan et al., 2019). It demonstrates that the absorption of CO_2_ of the membranes within such pressure range almost follows Henry's Law, and no effect due to compaction is observed.

## Conclusions

In summary, we fabricate a series of dual-filler MMMs by matching two non-2D fillers, SiO_2_ and HNTs, with two 2D fillers, GO and MXene, respectively. All dual-filler MMMs exhibit superior gas separation performance compared to the corresponding single filler MMMs, revealing the existence of synergetic effect between each pair of fillers. Such effect at low overall loading (1 wt%) is more notable than that at high loading (5 wt%), arising from the better dispersion of samples with 1 wt% filler. Interestingly, GO and MXene are found to meet different preferential partners due to their differences. On one hand, GO/HNTs proves to be a better pair than GO/SiO_2_, since HNTs are known to be wrapped by the flexible GO sheets so as to promote the dispersion of nanotubes. In turn, HNTs are deemed to hinder the restacking of GO sheets because of the strong steric effect. Compared to Pebax-HNTs and Pebax-GO membranes, the Pebax-GO/HNTs-0.5/0.5 membrane has optimal CO_2_ permeability with the enhancement of 107 and 100%, respectively. On the other hand, MXene works well with SiO_2_ rather than HNTs. In particular, the Pebax-MXene/ SiO_2_-0.2/0.8 membrane achieves 33% and 58% enhancement of CO_2_ permeability and CO_2_/N_2_ selectivity compared to Pebax-MXene membrane.

## Data Availability Statement

All datasets generated for this study are included in the article/Supplementary Material.

## Author Contributions

YL designed the study. JS and FS prepared and characterized fillers and membranes. XC performed the PALS analysis. FS, YL, SN, SW, ML, ZY, and JW conducted data analysis, figure drawing, and writing.

### Conflict of Interest

The authors declare that the research was conducted in the absence of any commercial or financial relationships that could be construed as a potential conflict of interest.

## Abbreviations

Si(OEt)_4_: tetraethyl orthosilicate
PSS: Polystyrene sulfonate sodium salt
silica-based MCM-41: ordered mesoporous silica spheres (MSSs) with MCM-41 type structure
g-C_3_N_4_: graphitic-phase carbon nitride
HKUST-1: a highly porous open-framework metal coordination polymer [Cu_3_(TMA)_2_(H_2_O)_3_]_n._
NH2-MIL-51: a porous metal-organic framework material comprising a bidentate organic compound bound by coordination to a metal ion, the metal ion being Al^III^ and the bidentate organic compound being 2,6-naphthalenedicarboxylate
JDF-L1: a layered microporous titanosilicate, also known as AM-1 and NTS
Pebax-A/B-X/Y: a MMMs with Pebax as the matrix and A and B as the filler, X and Y denote the wt% of filler A and B, respectively.
