# Supplementary material for: Exploration of the Synergy Between 2D Nanosheets and a Non-2D Filler in Mixed Matrix Membranes for Gas Separation
Source: Front Chem. 2020 Feb 5;8:58. doi: 10.3389/fchem.2020.00058 (PMC7013040; doi:10.3389/fchem.2020.00058)
Supplement: Supplementary file 1 [file Data_Sheet_1.docx]

Supplementary Material


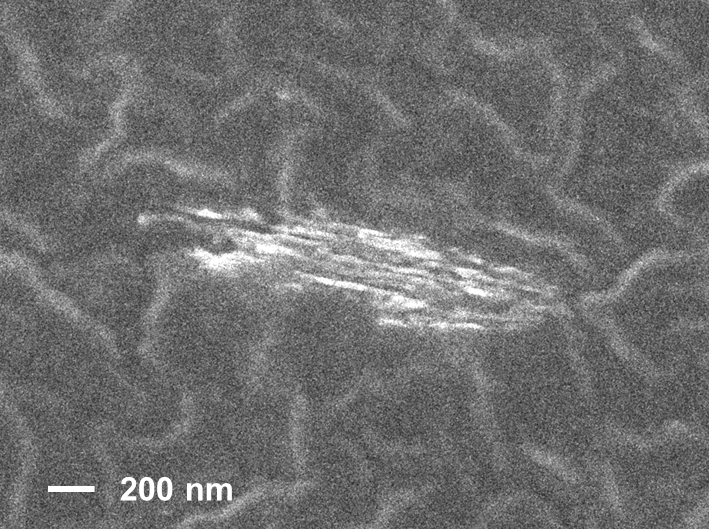


**FIGURE S1│**High-resolution cross-sectional SEM image of the typical aggregates of MXene in Pebax-MXene-5.


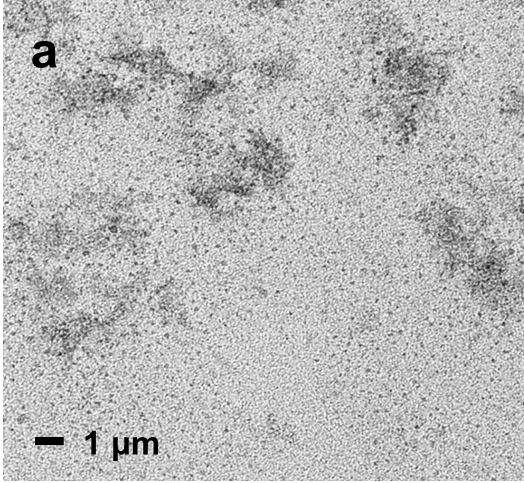

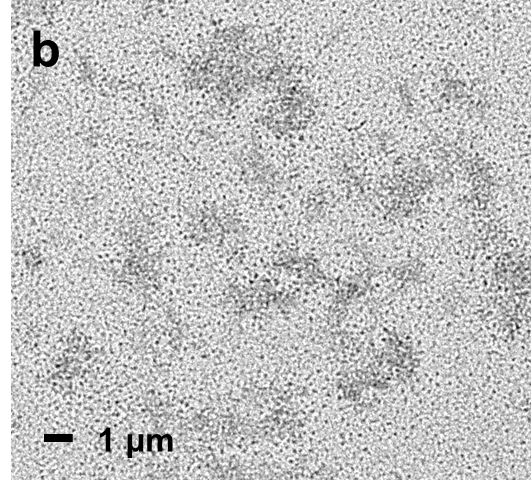


**FIGURE S2│**TEM images of the dispersion of MXene-SiO_2_ dual filler in Pebax membranes: (a) Pebax-SiO_2_/MXene-4/1 (b) Pebax-SiO_2_/MXene-0.8/0.2.


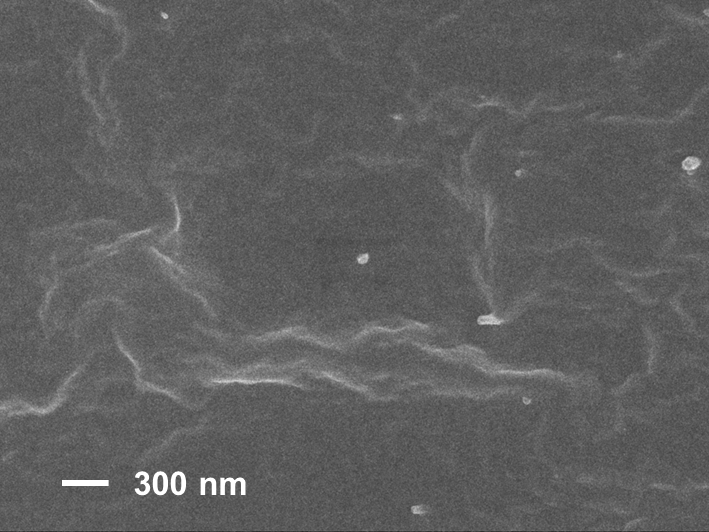


**FIGURE S3│**High-resolution cross-sectional SEM image of the filler dispersion in Pebax-GO/HNTs-0.5/0.5.


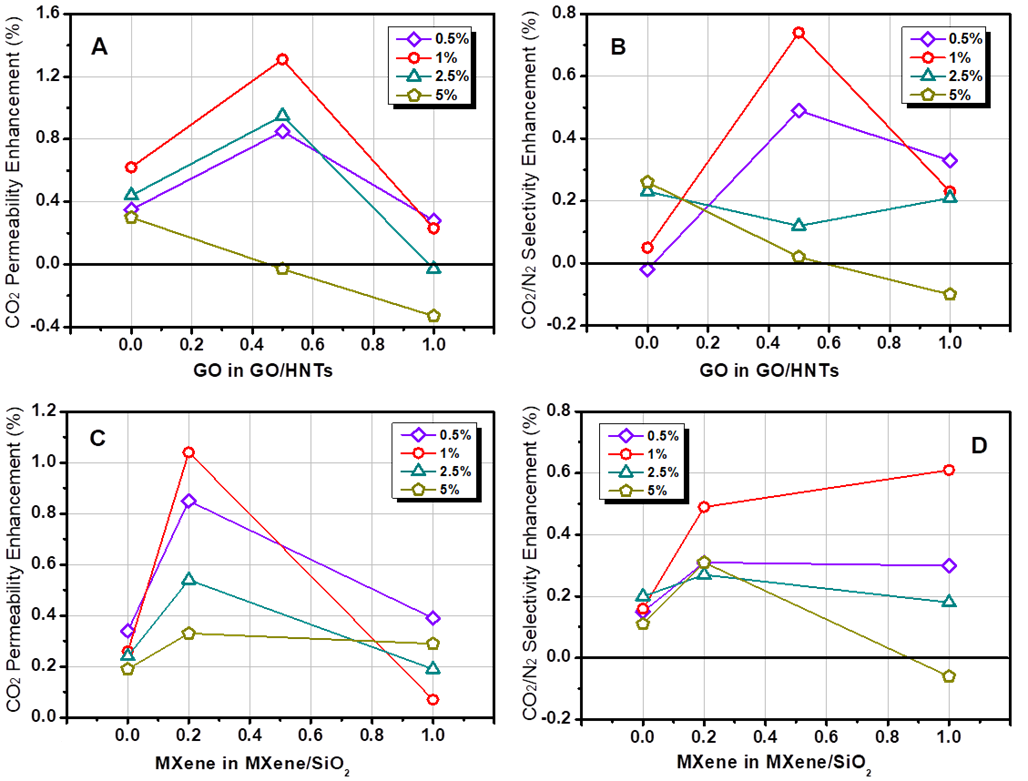


**FIGURE S4│**The effect of filler content on the gas transport properties of single and dual filler membranes.


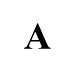

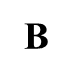


**FIGURE S5│**DSC curves of single and dual filler membranes: left-low temperature zone, right-high temperature zone.

**
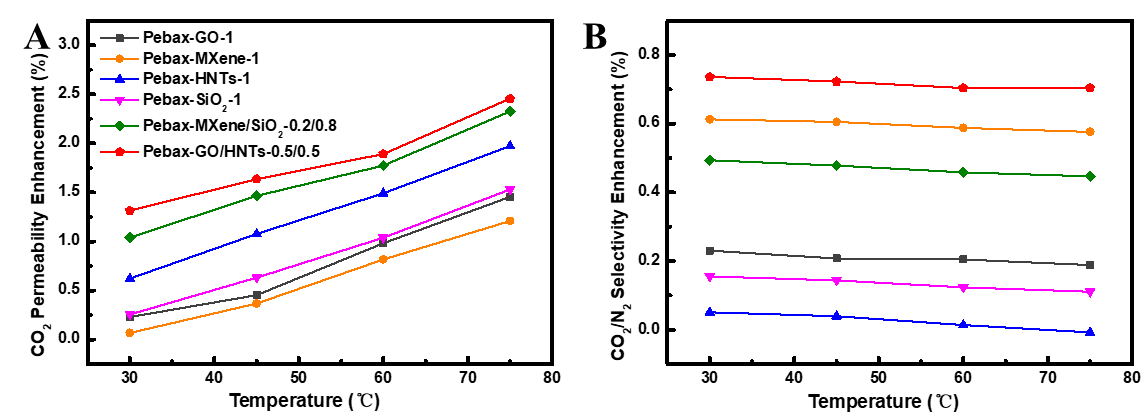
**

**FIGURE S6│**The effect of temperature on the gas transport properties of single and dual filler membranes.

**FIGURE S7│**Pure CO_2_ gas permeability as a function of reciprocal temperature.

**FIGURE S8│**The effect of pressure on the gas transport properties of single and dual filler membranes.

**Table S1│**Gas permeability, diffusivity and solubility coefficients of membranes.

| **Membrane** | ***D*_CO2_^a^** | ***D_CO2/_ D_N2_*** | ***S*_CO2_^b^** | ***S_CO2/_ S_N2_*** | ***P_CO2_^c^*** | ***P_CO2/_ P_N2_*** |
| --- | --- | --- | --- | --- | --- | --- |
| **Pebax** | 0.69 | 4.9 | 150 | 8.8 | 105 | 41 |
| **Pebax-GO-1** | 1.1 | 6.1 | 120 | 8.6 | 131 | 50 |
| **Pebax-SiO_2_ -1** | 1.3 | 11.8 | 100 | 5.6 | 133 | 47 |
| **Pebax-HNTs-1** | 3.2 | 12.8 | 54 | 3.4 | 171 | 43 |
| **Pebax-MXene-1** | 0.96 | 12.0 | 118 | 6.2 | 113 | 66 |
| **Pebax-SiO_2_/GO-0.2/0.8** | 1.5 | 8.8 | 113 | 5.9 | 171 | 52 |
| **Pebax-SiO_2_/GO-0.5/0.5** | 1.9 | 15.8 | 98 | 3.3 | 184 | 56 |
| **Pebax-SiO_2_/GO-0.8/0.2** | 1.7 | 14.2 | 98 | 3.1 | 166 | 43 |
| **Pebax-HNTs/GO-0.2/0.8** | 1.5 | 6.0 | 94 | 7.8 | 144 | 46 |
| **Pebax-HNTs/GO-0.5/0.5** | 3.2 | 7.1 | 76 | 6.3 | 245 | 71 |
| **Pebax-HNTs/GO-0.8/0.2** | 3.2 | 8.9 | 53 | 4.4 | 169 | 57 |
| **Pebax-SiO_2_/MXene-0.2/0.8** | 1.4 | 15.6 | 77 | 3.0 | 114 | 47 |
| **Pebax-SiO_2_/MXene-0.5/0.5** | 2.3 | 15.3 | 91 | 3.4 | 211 | 52 |
| **Pebax-SiO_2_/MXene-0.8/0.2** | 2.2 | 14.7 | 99 | 3.4 | 216 | 61 |
| **Pebax-HNTs/MXene-0.2/0.8** | 0.99 | 6.6 | 138 | 7.3 | 137 | 47 |
| **Pebax-HNTs/MXene-0.5/0.5** | 1.2 | 6.3 | 133 | 6.3 | 166 | 42 |
| **Pebax-HNTs/MXene-0.8/0.2** | 1.4 | 5.0 | 121 | 6.4 | 168 | 51 |

^a^ 10^-6^ cm^2^/s

^b^ 10^-4^ cm^3^ (STP)/cm^3^cmHg

^c.^ Barrer, 1 Barrer = 10^-10^ cm^3^ (STP) cm cm^-2^ s^-1^ cmHg^-1^

**Table S2│**Thermal properties of Pebax and corresponding mixed matrix membranes.

| **Membranes** | ***T*_g_** **(°C)** | **PEO** | | **PA6** | |
| --- | --- | --- | --- | --- | --- |
|  |  | ***T*_g_** **(°C)** | ***X*_c_ (%)** | ***T*_m_ (°C)** | ***X*_c_ (%)** |
| **Pebax** | -51.6 | 16.71 | 19.33 | 207.11 | 15.03 |
| **Pebax-HNTs-1** | -43.8 | 16.64 | 19.78 | 205.34 | 19.14 |
| **Pebax-GO-1** | -44.7 | 16.55 | 18.41 | 206.18 | 14.55 |
| **Pebax-SiO_2_-1** | -44.6 | 16.48 | 20.67 | 201.53 | 18.57 |
| **Pebax-MXene-1** | -48.7 | 16.55 | 19.08 | 205.91 | 18.24 |
| **Pebax-GO/HNTs-0.5/0.5** | -43.6 | 15.68 | 21.08 | 203.35 | 18.89 |
| **Pebax-MXene/SiO_2_-0.2/0.8** | -44.2 | 16.35 | 21.96 | 203.65 | 18.34 |

**Table S3│**Free volume properties of membranes.

| **Membrane** | ***I*_3_ (%)** | ***τ*_3_(ns)** | ***r*_3_(nm)** | **FFV (%)** |
| --- | --- | --- | --- | --- |
| **Pebax** | 14.64 | 2.336 | 0.314 | 1.896 |
| **Pebax-GO-1** | 16.26 | 2.363 | 0.316 | 2.150 |
| **Pebax-MXene-1** | 16.50 | 2.383 | 0.317 | 2.208 |
| **Pebax-HNTs-1** | 16.01 | 2.381 | 0.317 | 2.139 |
| **Pebax-SiO_2_-1** | 13.25 | 2.369 | 0.316 | 1.755 |
| **Pebax-GO/HNTs-0.5/0.5** | 14.59 | 2.302 | 0.311 | 1.835 |
| **Pebax-MXene/SiO_2_-0.2/0.8** | 13.20 | 2.370 | 0.316 | 1.749 |
